# Antegrade persufflation of porcine kidneys improves renal function after warm ischemia

**DOI:** 10.3389/frtra.2024.1420693

**Published:** 2024-08-22

**Authors:** Catherine Min, Jean-Philippe Galons, Ronald M. Lynch, Leah V. Steyn, Nicholas D. Price, Brad P. Weegman, Michael J. Taylor, Abhishek Pandey, Robert Harland, Diego Martin, David Besselsen, Charles W. Putnam, Klearchos K. Papas

**Affiliations:** ^1^ Department of Physiology, University of Arizona, Tucson, AZ, United States; ^2^ Department of Surgery, University of Arizona, Tucson, AZ, United States; ^3^ Department of Medical Imaging, University of Arizona, Tucson, AZ, United States; ^4^ Department of Biomedical Engineering, University of Arizona, Tucson, AZ, United States; ^5^ Department of Radiology, University of Minnesota, Minneapolis, MN, United States; ^6^ Sylvatica Biotech, Inc., North Charleston, SC, United States; ^7^ Department of Mechanical Engineering, Carnegie Mellon University, Pittsburgh, PA, United States; ^8^ Department of Electrical and Computer Engineering, University of Arizona, Tucson, AZ, United States; ^9^ University Animal Care, University of Arizona, Tucson, AZ, United States

**Keywords:** kidney assessment, organ preservation, oxygen consumption rate, persufflation, dynamic contrast-enhanced magnetic resonance imaging

## Abstract

**Introduction:**

Transplantation of kidneys from expanded criteria donors (ECD), including after circulatory death (DCD), is associated with a higher risk of adverse events compared to kidneys from standard criteria donors. In previous studies, improvements in renal transplant outcomes have been seen when kidneys were perfused with gaseous oxygen during preservation (persufflation, PSF). In the present study, we assessed ex-vivo renal function from a Diffusion Contrast Enhanced (DCE)-MRI estimation of glomerular filtration rate (eGFR); and metabolic sufficiency from whole-organ oxygen consumption (WOOCR) and lactate production rates.

**Methods:**

Using a porcine model of DCD, we assigned one kidney to antegrade PSF, and the contralateral kidney to static cold storage (SCS), both maintained for 24 h at 4°C. Post-preservation organ quality assessments, including eGFR, WOOCR and lactate production, were measured under cold perfusion conditions, and biopsies were subsequently taken for histopathological analysis.

**Results:**

A significantly higher eGFR (36.6 ± 12.1 vs. 11.8 ± 4.3 ml/min, *p* < 0.05), WOOCR (182 ± 33 vs. 132 ± 21 nmol/min*g, *p* < 0.05), and lower rates of lactate production were observed in persufflated kidneys. No overt morphological differences were observed between the two preservation methods.

**Conclusion:**

These data suggest that antegrade PSF is more effective in preserving renal function than conventional SCS. Further studies in large animal models of transplantation are required to investigate whether integration with PSF of WOOCR, eGFR or lactate production measurements before transplantation are predictive of post-transplantation renal function and clinical outcomes.

## 1 Introduction

Despite increased awareness, solicitation, and organization of organ donation in the United States and concerted efforts to expand the number of organs available for transplantation while balancing fair access (equity) and efficient use (utility) (1), the waiting list for kidney transplantation in the US now exceeds 90,000 persons. Recognizing the inadequacy of the supply of organs to meet current and future needs of the transplant recipient population, the transplant community continues to reassess the criteria for donor acceptability with the goal of increasing organ availability (2, 3). Consideration of kidneys from expanded criteria donors (ECD) and donations after circulatory death (DCD) perforce increase the latent organ pool. However, apprehension about the quality of ECD and DCD donations may cause transplant centers to reject kidneys because of their perceived higher rates of primary non-function (PNF) and delayed graft function (DGF), which jeopardize graft survival statistics, adversely affect the rates of patient morbidity and mortality, and compromise transplant center quality metrics.

Concerted efforts to advance organ preservation notwithstanding, the principal methodology in use for deceased donor kidney preservation remains static cold storage (SCS), in large part because of its simplicity and its long history of success in the realm of donation after brain death (DBD). Because of the predominance of intraregional organ sharing (4), cold ischemia times are usually less than 24 h, which is supportable by SCS. With DCD, however, an additional variable affecting organ quality is quite obviously introduced: an interval of warm ischemia time (WIT) which is difficult to precisely quantify as to extent and duration. A common if unwritten criterion of many transplant centers for refusal of DCD kidneys is a warm ischemia time estimated to be greater than 45–60 min (5).

Although SCS quickly slows metabolism by rapid cooling of the renal parenchyma (6), prevenient warm ischemia results in diminished energy reserves which continue to be consumed during WIT without replenishment. Adenosine triphosphate (ATP) stores likewise cannot be renewed during subsequent hypoxic SCS. Various approaches for machine perfusion have been explored in efforts to improve the quality of donated organs which have suffered significant warm ischemia; these include hypothermic machine perfusion (HMP) and normothermic machine perfusion (NMP) and numerous modifications thereof, as reviewed in (7–10). Oxygenation of the perfusate can be integrated into HMP but is constrained by the solubility of gas in the water-based preservation solution. Oxygenation is an essential component of NMP in which the perfusate includes erythrocytes or oxygen-carrying blood substitutes. The premise is simple: providing oxygen to the kidney enables aerobic metabolism, facilitating replenishment of depleted ATP stores, and thus “resuscitating” the kidney by ameliorating or reversing deleterious effects of warm ischemia. Moreover, if perfused with sufficient oxygen, even organs maintained under hypothermic conditions will continue to produce and utilize energy (8, 9, 10, 11).

An alternative approach to preservation by SCS, HMP or NMP is persufflation (PSF): the organ vasculature is perfused, not with a liquid perfusate, but by humidified air supplemented with oxygen at a supra-ambient partial pressure of oxygen (PO2) [reviewed in (11–13)]. However, PSF has failed to gain traction in organ preservation, despite a number of reports demonstrating its experimental effectiveness in the preservation of various organs, including the heart (14, 15), lung (16), liver (15, 17, 18), pancreas (19–21) and kidney (13, 17, 22). Of note, in animal models of DCD, PSF has a demonstrably beneficial effect upon organ function (23–27).

In the early studies of kidney PSF, investigators pursued both the “antegrade” (*via* the renal artery) and the “retrograde” (*via* the renal vein) approaches, as previously reviewed in detail (13). However, retrograde PSF of the kidney requires creating multiple tiny puncture wounds in the capsule to relieve pressure; antegrade PSF, on the other hand, has the perceived disadvantage of exposing the endothelium of the arterial system to higher gas pressures. In the early 1970's, studies by Isselhard, comparing the two approaches, concluded that better preservation was achieved with retrograde PSF (28–32). Based on Isselhard's work, the retrograde approach dominated renal persufflation thereafter. However, because of clinicians' concerns that even minor injury to the organ by needle pricks could potentially lead to complications during the early post-transplantation period, and recent technical refinements in devices to deliver PSF, we chose in the current study to evaluate the more physiologic antegrade approach to persufflation.

A difficulty encountered in any animal study of organ preservation is the dearth of *ex vivo* real time analytics of proven value for predicting either PNF or DGF. Although uncommonly exploited for this purpose, a body of evidence indicates that oxygen consumption rate (OCR) is a reliable method for assessing metabolic activity, hence viability, of isolated pancreatic islets (33–37) and intact kidneys (whole organ OCR, WOOCR) (38, 39). We therefore chose WOOCR as a measure of global metabolic activity of the preserved kidney. A second methodology that we applied to evaluate the efficacy of PSF on renal function in real time is dynamic contrast-enhanced magnetic resonance imaging (DCE-MRI), a non-invasive technique for the visualization of intrarenal blood flow; importantly, it can discriminate between cortical and medullary blood flow (40–43). Data derived from DCE-MRI can be used to calculate certain physiological parameters, including regional filtration, filtration fraction and estimated glomerular filtration rate (eGFR), which are recognized measures of kidney function.

In the current studies we used a DCD porcine kidney model, integrating oxygen-sensing (OCR) and imaging technology (DCE-MRI), a conventional metabolic parameter (lactate production), and histopathology. WOOCR, DCE-MRI-estimated eGFR and lactate production were measured *ex vivo* in kidneys subjected to 30 min of WIT and 24 h of cold ischemia time (CIT) to mimic DCD clinical renal transplantation. Under these conditions and based on the specified parameters, we conclude that PSF preservation is superior to SCS.

## 2 Materials and methods

### 2.1 Animal ethics

This study was approved by the institutional animal care and use committee (IACUC) of the University of Arizona. All animal care procedures were in accordance with university policies. Domestic female juvenile pigs weighing 35–50 kg were purchased from Premier BioSource (www.premierbiosource.com).

### 2.2 Organ procurement and preservation

**Figure 1:**
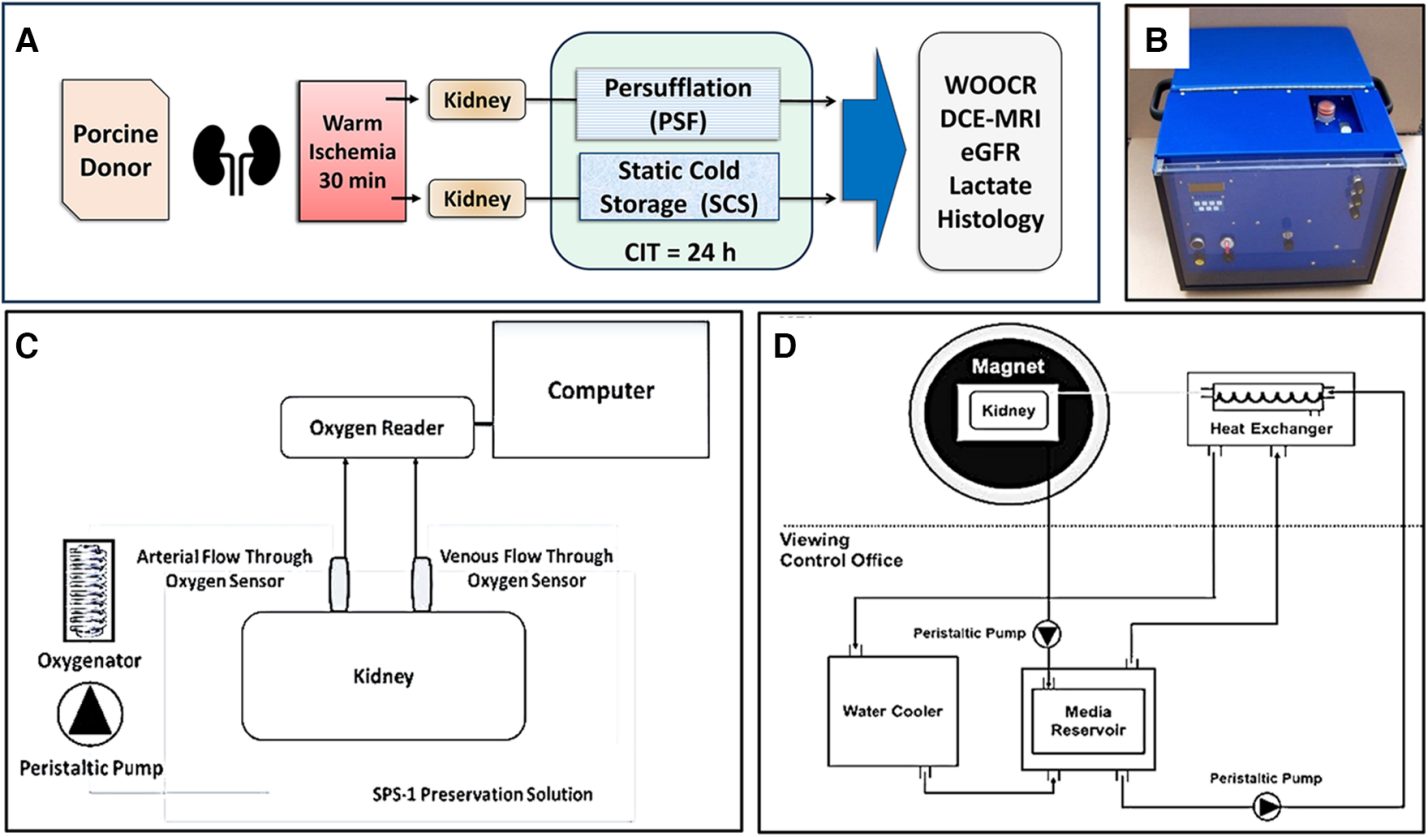
Schematic illustrations of the methodology. **(A)** Experimental design. Paired porcine kidneys were procured after 30 min WIT, then preserved for 24 h, either by SCS or PSF, for 24 h of CIT. Measures of renal function were assessed after concluding preservation. **(B)** A self-contained kidney persufflation system; within the case is the oxygen generator and a container for the kidney. The gas (40% oxygen) pressure is maintained at 40–50 mmHg) by the unit. **(C)** Experimental measurement of WOOCR. The partial pressure of oxygen (PO2) was measured with oxygen sensors positioned at the input and output ports of a liquid perfusion circuit. WOOCR values were calculated from the differential of the arterial and venous PO2 readings, the flow rate, and oxygen solubility at the measured temperature, normalized to the mass of the kidney. **(D)** Experimental evaluation of renal parenchymal perfusion by DCE-MRI. Images were obtained from isolated porcine kidneys after 24 h CIT, using an MRI-compatible hypothermic perfusion system.

The experimental design is depicted in Figure 1A. Briefly, heparinized porcine donors were euthanized, exsanguinated, and their circulation flushed with two liters of heparinized saline solution at room temperature via the abdominal aorta. The kidneys (and other organs) then remained in place for 30 min of warm ischemia. After bilateral nephrectomy, the renal arteries and veins were cannulated with luer-lock catheters (Cole-Parmer). The kidneys were initially flushed with 400 ml of 4°C heparinized saline solution, followed by a second flush with SPS-1 solution (Organ Recovery Systems). One of the two kidneys was randomized to PSF vs. SCS; the other kidney was by default assigned to the alternate method. The prototype persufflation device (Giner, Inc., Newton, MA) delivers electrochemically generated 40% oxygen mixed with atmospheric air; the gas stream is humidified (Figure 1B). The persufflator maintains a manifold pressure of 40–50 mmHg which generates a gas flow rate of ∼60 ml/min. The gas outflow tube of the persufflator is attached to the renal arterial cannula and any gas leaks from the tissues surrounding the vessels are ligated. Both kidneys – PSF and SCS – were placed in SPS-1 solution at 4°C during the 24 h of preservation.

### 2.3 Renal oxygenated HMP and whole organ oxygen consumption rate (WOOCR)

At the conclusion of the 24 h period of either PSF or SCS preservation, the previously cannulated renal artery and vein were connected to a custom-built hypothermic machine perfusion (HMP) system (Figure 1C) maintained at a temperature of 4 to 8°C. SPS-1 solution was oxygenated with 40% oxygen using a hollow-fiber oxygenator (Medtronic, Minneapolis, MN) and the kidney was perfused via the artery during the OCR measurement. Flow-through fiber optic oxygen sensors (Instech Laboratories, Inc., Plymouth Meeting, PA) were installed just proximal to the arterial and distal to the venous cannulae to measure the decrement of the partial pressure of oxygen (PO2) across the organ. Before any experimental measurements were taken, the inline sensors were calibrated at 0%, 21% and 40% oxygen. The WOOCR (mol/min/g) of the kidney was calculated by using the mass balance equation (below), in which *Q* is the (perfusion) flow rate in ml/min, *α* is the solubility (mol/ml/mmHg) of oxygen in an aqueous solution at the given temperature, and *m* is the mass of the kidney in g (39):

$$\mathrm{WOOCR}=\frac{Q\times (\;pO_{2\;\mathrm{Arterial}}-pO_{2\;\mathrm{Venous}})\times \alpha }{m}$$

### 2.4 Dynamic contrast-enhanced magnetic resonance imaging (DCE-MRI)

Imaging was performed with a 3T Skyra scanner (Siemens). The kidneys were secured in a repurposed human kidney cassette (Organ Recovery Systems, Itasca, IL) and cold-perfused (7°C) with SPS-1 preservation solution, delivered via a MR-compatible perfusion circuit. The kidney cassette was inserted into a standard Siemens Head and Neck RF coil (Figure 1D) After 20 s of pre-contrast image acquisition, the MRI contrast medium, a 4 mM solution of Multihance Gadolinium (Gd)-DTPA (Bracco Diagnostics Inc. Monroe Township, NJ) was injected into the cannulated artery. Continuously acquired images were recorded for 4 min. DCE-MRI was performed using Siemens' golden angle stack of star radial acquisition (radialVibe). Compressed sensing reconstruction with total variation applied in the temporal dimension was used to reconstruct images with 1 s temporal resolution. The two-compartment model proposed by Tofts (43) was used to estimate GFR from DCE-MRI. The arterial input function (AIF) was calculated from the signal acquired from the renal artery. The signal intensity-time curve for the entire kidney was converted to a concentration-time curve using MultiHance R1 (relaxivity) and T1 relaxation times for the SPS-1 solution measured at 7°C. A delayed mono-exponential function was used to model delay (*τ*) and dispersion (*d*) of the AIF signal as the perfusate reached the renal parenchymal capillary bed. The nonlinear least square fit method was used to extract four kinetic parameters: *K_trans_*, *Vb*, *τ*, and *d*. The transfer constant *K_trans_* provides a measure of filtration of Gd contrast from the intra-vascular (IV) to the extra-vascular (EV) space; *Vb* is the fraction of liquid within the renal vasculature. From the mean *K_trans_* obtained from the kidney signal, the total filtration in the parenchyma can be calculated as:

$$\mathrm{GFR}\;=K_{trans}\;\times \;\mathrm{Kidney}\;\mathrm{volume}.$$

### 2.5 Lactate measurements

After 24 h of preservation, kidneys were liquid perfused through the renal artery with SPS-1 solution in a recirculating hypothermic circuit maintained at 4–8°C. Kidneys were perfused at a flow rate of 80 ml/min, and the initial 500 ml of effluent from the renal vein was collected for analysis of lactate content. The amount of lactate measured in the initial 500 ml of the recirculation medium closely approximates the amount of lactate released from cells of the kidney during the 24-hour preservation period. Effluent lactate concentration was measured with the YSI 2700 biochemistry analyzer (YSI Life Sciences) and normalized to the renal mass.

### 2.6 Histopathology

Wedge biopsies were taken from the kidneys after the evaluation period following preservation. They were fixed with 4% paraformaldehyde, dehydrated, processed and embedded in paraffin. Tissue samples were sectioned at 5 μm with a microtome and stained with hematoxylin and eosin (H&E) (Sigma-Aldrich, St. Louis, MO) to evaluate renal morphology by light microscopy. The sections were assessed for tubular, glomerular and vascular damage at 100×, 200× and 400×. A veterinary pathologist (D.B.) blinded to the treatment groups estimated the amount of damage per tissue section, scored over 5 fields per slide, using a 4-point scale in which 0 indicates no abnormalities; 1, mild changes; 2, moderate changes; and 3, severe changes.

### 2.7 Statistics

The data of *WOOCR*, *eGFR,* *K_trans_* and lactate production were all accrued from paired kidneys; therefore, the differences between the treatment groups were analyzed using a paired *t*-test, run on Graphpad Prism. Statistical significance was set at a *P* value ≤ 0.05. Unless otherwise indicated, the mean and SEM values are plotted.

## 3 Results

### 3.1 Whole organ oxygen consumption rate (WOOCR)

**Figure 2:**
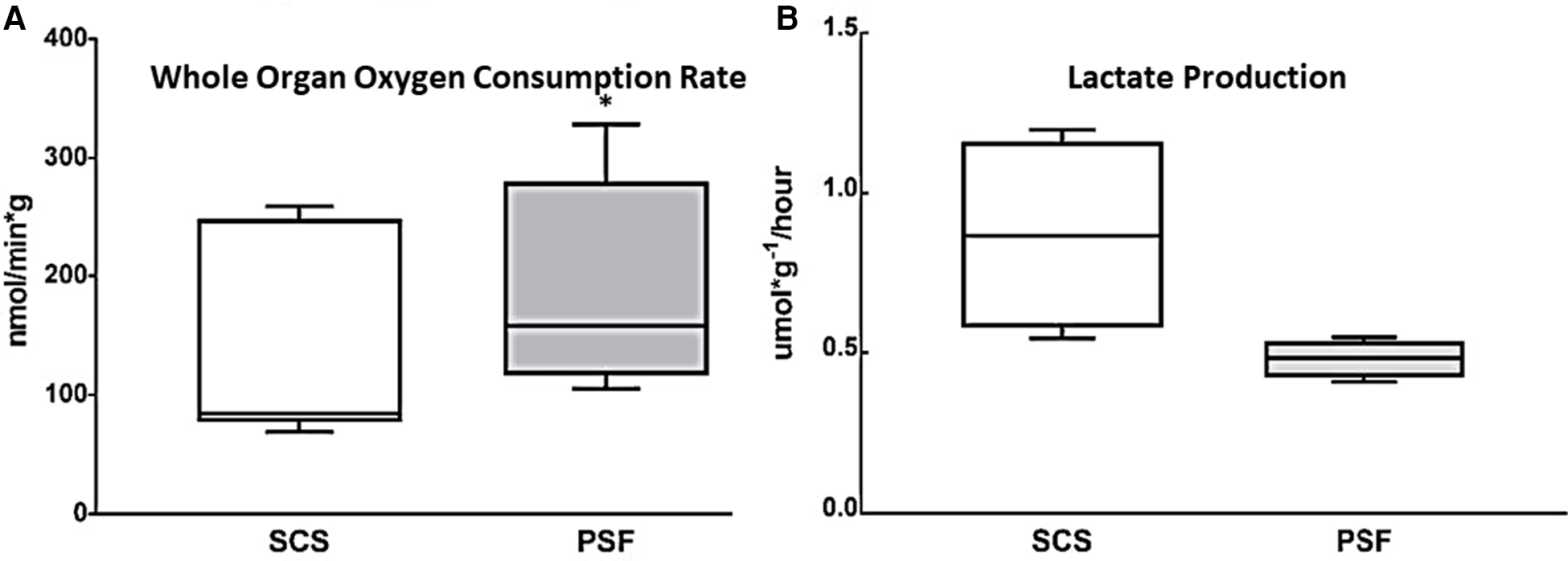
Whole kidney oxygen consumption and lactate production after 30 min WIT and 24 h CIT, during which preservation was with either SCS or PSF. **(A)** WOOCR, measured as shown in Figure 1C, was significantly greater after PSF compared to SCS (*n* = 7, * *p* < 0.05). **(B)** Lactate production normalized to kidney mass over 24 h of preservation at 4°C, as calculated from lactate concentration measured in the initial 500 ml effluent collected after preservation, was significantly (*p* ≤ 0.05) greater after SCS than PSF. Shown are box and whisker plots displaying the minimum score, first (lower) quartile, median, third (upper) quartile, and maximum score.

To assess global metabolic activity, WOOCR was evaluated after 30 min of WIT followed by 24 h of cold preservation time (CPT), in paired kidneys subjected to either PSF or SCS (*n* = 7 kidneys per group). When measured at 4–8°C, mean WOOCR in PSF kidneys was 182 (SEM 33) nmol/min/g vs. 132 (SEM 21) nmol/min/g in SCS kidneys, a significant (*p* < 0.05) difference (Figure 2A). That persufflated kidneys consumed oxygen at a greater rate indicates, globally, a more active metabolism.

### 3.2 Lactate production

Lactate, a product of anaerobic metabolism, is elevated when aerobic metabolism has failed. It was measured in effluent collected from the isolated kidney immediately following the 24-h preservation period. The amount of accumulated lactate was compared for paired kidneys (*n* = 4) subjected to either SCS or PSF (Figure 2B). The concentration of lactate in the initial wash of 500 ml of preservation solution and normalized to renal mass was nearly double for static cold storage kidneys, 20.8 (SEM 7) μmol/g, as for persufflated kidneys, 11.5 (SEM 1.4) μmol/g, a statistically significant difference. Additionally, the rate of lactate production over the 24 h of cold preservation was calculated (Figure 2B). Renal tissue, if supplied with oxygen, metabolizes lactate by gluconeogenesis; therefore, the lower lactate concentration in the eluate from PSF-preserved kidneys may be the consequence of decreased production, increased utilization, or, most likely, both.

### 3.3 Dynamic contrast enhanced magnetic resonance imaging (DCE-MRI)

**Figure 3:**
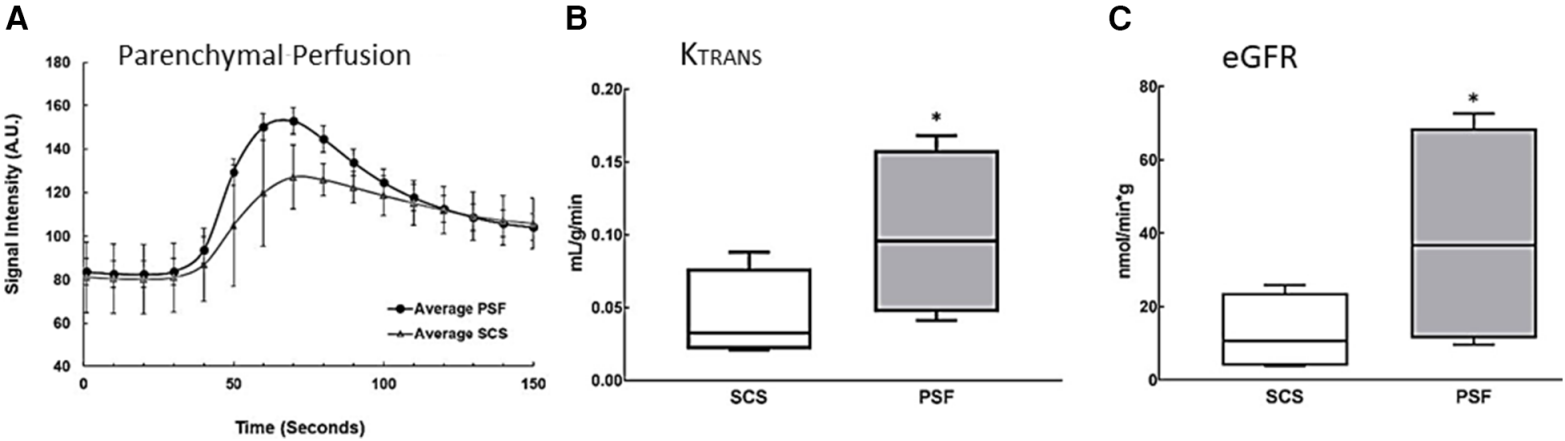
Estimation of functional parameters *K_trans_* and *eGFR* from renal perfusion data obtained by DCE-MRI. Images were obtained from isolated porcine kidneys preserved for 24 h using an MRI-compatible hypothermic perfusion system, see Figure 1D. **(A)** DCE-MRI perfusion curves. Regions of interest around the entire kidney were imaged and graphed to show the average signal intensity over time in the renal cortex and medulla. The descending slope represents clearance of tracer from the renal cortex; it is significantly steeper in kidneys preserved with PSF (*n* = 4, *p* ≤ 0.05). **(B)** *K_trans_*, the transfer constant of Gd moving from the intravascular (glomerular) space into the extravascular, (tubular space), was higher in PSF-preserved kidneys than in SCS-preserved kidneys (*n* = 5, *p* ≤ 0.05). **(C)** *eGFR* was estimated from *K_trans_* and total kidney volume. The *eGFR* was significantly higher (*n* = 5, *p* ≤ 0.05) in PSF than SCS kidneys, supporting the more favorable efficacy of PSF as an organ preservation method. The box plots are described in the legend of Figure 2.

Although the *global* metabolic activity of PSF-preserved kidneys was greater than that of SCS-preserved organs, differences in local renal perfusion and thus in GFR could negate that advantage. DCE-MRI was therefore evaluated in five pairs of kidneys after the 24-h preservation period was completed; Gd was the contrast agent. Parenchymal perfusion curves showed different slopes for the two preservation methods (Figure 3A). SCS kidneys exhibited a descending cortical slope of −0.40 ± 0.30, whereas the DCE-MRI of PSF kidneys rendered a higher peak and a significantly steeper slope of −1.38 (SEM 0.34), indicating significantly (*p* < 0.05) greater perfusion and a faster clearance rate of contrast from the renal cortex.

*K_trans_* (see Materials and Methods) reflects the efflux rate of Gd from the intravascular to the extravascular space, which in the kidney is almost exclusively engendered by glomerular (intravascular space) filtration into the renal tubules (extravascular space). SCS kidneys had a mean *K_trans_* value of 0.04 ± 0.01 ml/g/min while persufflated kidneys exhibited a nearly three-fold higher rate of 0.11 (SEM 0.02) ml/g/min (Figure 3B). From *K_trans_* values derived from data acquired at 7°C, eGFR was calculated (normalized to the renal volume), Figure 3C. Persufflated kidneys (PSF) exhibited a significantly higher eGFR of 36.6 (SEM12.1) ml/min than the SCS kidneys, eGFR of 11.8 (SEM 4.3) ml/min). In sum, the DCE-MRI documented superior cortical perfusion and glomerular function of the persufflated kidneys, in comparison to matched kidneys submitted to SCS.

### 3.4 Histology

**Figure 4:**
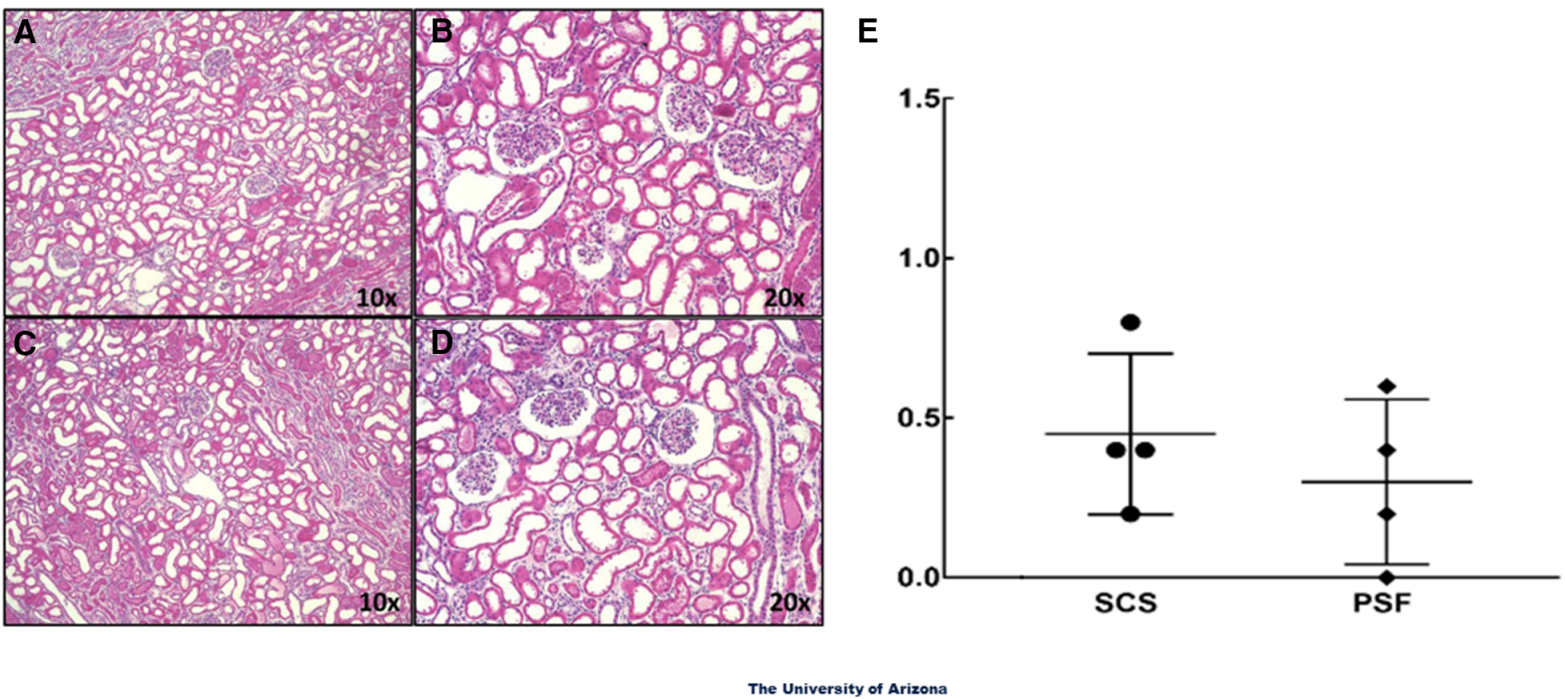
Light microscopic images of H&E sections of renal tissue samples. Wedge biopsies of the renal cortex were taken from isolated DCD kidneys after the 24-hour preservation period. **(A)** and **(B)** are images from PSF kidneys and **(C)** and **(D)** from SCS kidneys. The glomeruli and renal vessels in the biopsies appeared intact in most areas examined. No overt signs of structural damage were observed in either experimental group. The magnifications noted are of the objective only; the total magnifications are 100× and 200×. **(E)** Summation of the light microscopic evaluations. Data presented are average scores from 5 fields of view from each kidney section for 4 different kidneys in each treatment group.

Even though PSF-preserved kidneys had superior functional parameters by WOOCR and DEC-MRI, histological evaluation failed to reveal any significant differences in morphology between the SCS and PSF kidneys; both exhibited mild vacuolation and scattered pyknotic nuclei, which are early signs of degeneration and apoptosis (Figures 4A-D). Neither group exhibited any notable damage to the renal vasculature. Based on the pathological scores, there were no significant differences between the two groups, with minimal damage to renal morphology observed in the renal biopsies of both groups (Figure 4E). The histologic similarities of pairs of kidneys submitted to PSF or SCS were not unanticipated; much of the tissue damage from WIT + CPT only becomes histologically evident after restoring normal circulation (reperfusion injury) by transplantation.

## 4 Discussion

Our principal objective in this study was to evaluate the potential of PSF of kidneys to potentially (1) improve the quality and extend the duration of permissible organ preservation; and (2) mitigate damage from warm ischemia inflicted on the kidney in the donor before organ removal. These objectives are shared by hypothermic machine perfusion (especially for ECD and DCD organs), which is gaining traction for renal preservation. However, static cold storage/transport remains the dominant clinical preservation modality today. Thus, SCS was chosen to represent current clinical practice for our studies.

This report, and the data herein, address three considerations relevant to the current climate of kidney transplantation from deceased donors, especially DCD organs. First, persufflation with 40% oxygen gas proved superior to static cold storage, in a direct comparison of kidney pairs from single donors submitted simultaneously to 30 min of warm ischemia, followed by equal intervals (24 h) of cold preservation. Secondly, the increased OCR (38, 39) and decreased lactate production (44–49) of PSF kidneys compared to their SCS counterparts provided evidence of enhanced metabolism attributable to the effective delivery of oxygen; these data also suggest that oxygen-dependent gluconeogenesis within PSF kidneys might have contributed to decreased measured lactate production by consuming lactate during PSF (44–49). Thirdly, although the kidneys were not tested by transplantation, increased cortical perfusion, faster clearance from the cortex of Gd MRI contrast, and increased *K_trans_* and its derivative, (*eGFR*), attest to the likelihood of superior functionality of the PSF kidneys. These observations are most readily unified by the notion that PSF reversed at least some of the damage inflicted by the earlier period of warm ischemia.

A fourth issue beleaguering the utilization of DCD kidneys was not directly addressed in this study, namely, the identification of parameters predicting the functional viability of the organ in its recipient. If a kidney preordained to be permanently non-functional (PNF) can be *reliably* identified before transplantation, it could be declined, instead of determining its viability by implanting it in a patient. The antithetical dilemma, a prediction of *delayed graft function* should not preclude transplantation; additionally, a well-grounded prognosis could inform subsequent clinical decision-making, *e.g.,* criteria for specific recipient selection; early management of immunosuppression; and the avoidance of invasive diagnostic interventions. Currently, measurements of vascular resistance (45) during HMP or NMP are considered useful indicators but are not discriminating predictors of PNF or DGF; histopathological assessments of pre-transplantation biopsies (46, 47) are also useful but require special expertise to accurately interpret and may not be available before making a transplant decision. The data in this report suggest the possibility – but did not definitively test the hypothesis by transplantation – that organ OCR and lactate production might fulfil this objective (DCE-MRI would also be useful but is logistically problematic). It will be important to determine the sensitivity, specificity, and the area under the curve (AUC) of the receiver operating characteristic (ROC), of OCR and lactate production for the prediction of PNF and DGF; these determinations require testing by transplantation PSF-preserved (pig) kidneys after WITs of increasing durations. Additionally, comparison of real-time WOOCR measurements with standard static markers of acute kidney injury (AKI), such as neutrophil gelatinase-associated lipocalin (NGAL), and kidney injury molecule-1 (KIM-1) (50, 51), may refine the sensitivity of predictive renal function analysis prior to transplantation. Establishing and verifying reliable predictive parameters would also facilitate investigations of additional, complementary organ preservation interventions, such as the inclusion of drugs or biologics in the persufflate gas mixture.

The data herein support an earlier report that antegrade persufflation of the kidney provides effective preservation. We did not however directly compare antegrade to retrograde PSF. Higher gas pressures (60–100 mmHg) were employed in early antegrade studies (29, 30) and likely inflicted damage, accounting for poor function after transplantation. We chose the tenable hypothesis that the antegrade approach is innately more physiological; however, we carefully monitored and maintained a lower pressure (40 mmHg) than used earlier. It should be noted that for preservation of the pancreas by PSF for islet isolation, the antegrade rather than the retrograde route is usually, but not always, exploited; both appear satisfactory. Similarly, both routes have been explored for hepatic PSF (cannulating the vena cava for the retrograde approach; for antegrade PSF, either the portal vein and/or the hepatic artery has been used). Neither route enjoys a clear advantage.

## Data Availability

The original contributions presented in the study are included in the article/Supplementary Material, further inquiries can be directed to the corresponding author.

## References

[1] Bradbrook K Gauntt K Klassen D. Renal transplantation: the last iteration of the rest of the world. Curr Opin Organ Transplant. (2023) 28(3):207–11. 10.1097/mot.0000000000001059 36995686

[2] Rijkse E Ceuppens S Qi H IJzermans NMIJ Hesselink DA Minnee RC. Implementation of donation after circulatory death kidney transplantation can safely enlarge the donor pool: a systematic review and meta-analysis. Int J Surg. (2021) 92:106021. 10.1016/j.ijsu.2021.106021 34256169

[3] Croome KP Barbas AS Whitson B Zarrinpar A Taner T Lo D American Society of transplant surgeons recommendations on best practices in donation after circulatory death organ procurement. Am J Transplant. (2023) 23(2):171–9. 10.1016/j.ajt.2022.10.009 36695685

[4] Cron DC Husain SA King KL Mohan S Adler JT. Increased volume of organ offers and decreased efficiency of kidney placement under circle-based kidney allocation. Am J Transplant. (2023) 23(8):1209–20. 10.1016/j.ajt.2023.05.005 37196709 PMC10527286

[5] Foley ME Vinson AJ Skinner TAA Kiberd BA Tennankore KK. The impact of combined warm and cold ischemia time on post-transplant outcomes. Can J Kidney Health Dis. (2023) 10:20543581231178960. 10.1177/20543581231178960 37333478 PMC10272701

[6] Chen Y Shi J Xia TC Xu R He X Xia Y. Preservation solutions for kidney transplantation: history, advances and mechanisms. Cell Transplant. (2019) 28(12):1472–89. 10.1177/0963689719872699 31450971 PMC6923544

[7] Kaths JM Paul A Robinson LA Selzner M. Ex vivo machine perfusion for renal graft preservation. Transplant Rev (Orlando). (2018) 32(1):1–9. 10.1016/j.trre.2017.04.002 28483273

[8] Ghoneima AS Sousa Da Silva RX Gosteli MA Barlow AD Kron P. Outcomes of kidney perfusion techniques in transplantation from deceased donors: a systematic review and meta-analysis. J Clin Med. (2023) 12(12):3871. 10.3390/jcm12123871 37373568 PMC10298857

[9] Radajewska A Krzywonos-Zawadzka A Bil-Lula I. Recent methods of kidney storage and therapeutic possibilities of transplant kidney. Biomedicines. (2022) 10(5):1013. 10.3390/biomedicines10051013 35625750 PMC9139114

[10] Zulpaite R Miknevicius P Leber B Strupas K Stiegler P Schemmer P. Ex-vivo kidney machine perfusion: therapeutic potential. Front Med (Lausanne). (2021) 8:808719. 10.3389/fmed.2021.808719 35004787 PMC8741203

[11] Mesnard B Ogbemudia AE Karam G Dengu F Hackim G Rigaud J What is the evidence for oxygenation during kidney preservation for transplantation in 2021? A scoping review. World J Urol. (2022) 40(9):2141–52. 10.1007/s00345-021-03757-8 34432136

[12] Min CG Papas KK. Recent developments in persufflation for organ preservation. Curr Opin Organ Transplant. (2018) 23(3):330–5. 10.1097/mot.0000000000000526 29634496

[13] Suszynski TM Rizzari MD Scott WE 3rd Tempelman LA Taylor MJ Papas KK. Persufflation (or gaseous oxygen perfusion) as a method of organ preservation. Cryobiology. (2012) 64(3):125–43. 10.1016/j.cryobiol.2012.01.007 22301419 PMC3519283

[14] Minasian SM Galagudza MM Dmitriev YV Karpov AA Vlasov TD. Preservation of the donor heart: from basic science to clinical studies. Interact Cardiovasc Thorac Surg. (2015) 20(4):510–9. 10.1093/icvts/ivu432 25538253

[15] Suszynski TM Rizzari MD Scott WE Eckman PM Fonger JD John R Persufflation (gaseous oxygen perfusion) as a method of heart preservation. J Cardiothorac Surg. (2013) 8:105. 10.1186/1749-8090-8-105 23607734 PMC3639186

[16] Isselhard W Minor T. Gaseous oxygen for protection and conditioning of organs during ischemia. Zentralbl Chir. (1999) 124(4):252–9. 10355078

[17] Lee CY Mangino MJ. Preservation methods for kidney and liver. Organogenesis. (2009) 5(3):105–12. 10.4161/org.5.3.9582 20046672 PMC2781089

[18] Mahboub P Bozorgzadeh A Martins PN. Potential approaches to improve the outcomes of donation after cardiac death liver grafts. World J Transplant. (2016) 6(2):314–20. 10.5500/wjt.v6.i2.314 27358776 PMC4919735

[19] Kelly AC Suszynski TM Papas KK. Chapter 7 - oxygenation of the pancreas. In: Hawthorne WJ, editor. Pancreas and Beta Cell Replacement. 1st ed. Cambridge, MA: Academic Press (2022). p. 113–24.

[20] Reddy MS Carter N Cunningham A Shaw J Talbot D. Portal venous oxygen persufflation of the donation after cardiac death pancreas in a rat model is superior to static cold storage and hypothermic machine perfusion. Transpl Int. (2014) 27(6):634–9. 10.1111/tri.12313 24628941

[21] Scott WE 3rd O'Brien TD Ferrer-Fabrega J Avgoustiniatos ES Weegman BP Anazawa T Persufflation improves pancreas preservation when compared with the two-layer method. Transplant Proc. (2010) 42(6):2016–9. 10.1016/j.transproceed.2010.05.092 20692396 PMC2956134

[22] Hosgood SA Nicholson HF Nicholson ML. Oxygenated kidney preservation techniques. Transplantation. (2012) 93(5):455–9. 10.1097/TP.0b013e3182412b34 22217529

[23] Kageyama S Yagi S Tanaka H Saito S Nagai K Hata K Graft reconditioning with nitric oxide gas in rat liver transplantation from cardiac death donors. Transplantation. (2014) 97(6):618–25. 10.1097/tp.0000000000000025 24521773

[24] Koetting M Dombrowski F Minor T. No synergistic effect of carbon monoxide and oxygen during static gaseous persufflation preservation of dcd livers. J Surg Res. (2011) 171(2):859–64. 10.1016/j.jss.2010.06.009 20850768

[25] Kröger N Czigany Z Jiang J Afify M Paschenda P Nagai K The benefits of fibrinolysis combined with venous systemic oxygen persufflation (vsop) in a rat model of donation after circulatory death and orthotopic liver transplantation. Int J Mol Sci. (2022) 23(9):5272. 10.3390/ijms23095272 35563662 PMC9099893

[26] Porschen A Kadaba Srinivasan P Iwasaki J Afify M Tolba RH. Optimal timing for venous systemic oxygen persufflation supplemented with nitric oxide gas in cold-stored, warm ischemia-damaged experimental liver grafts. Eur Surg Res. (2016) 57(1–2):100–10. 10.1159/000445682 27271697

[27] Mownah OA Khurram MA Ray C Kanwar A Stamp S Rees D Development of an ex vivo technique to achieve reanimation of hearts sourced from a porcine donation after circulatory death model. J Surg Res. (2014) 189(2):326–34. 10.1016/j.jss.2014.02.041 24694717

[28] Isselhard W Berger M Denecke H Witte J Fischer JH Molzberger H. Metabolism of canine kidneys in anaerobic ischemia and in aerobic ischemia by persufflation with gaseous oxygen. Pflugers Arch. (1972) 337(2):87–106. 10.1007/bf00587833 4675076

[29] Isselhard W Denecke H Stelter W Berger M Sachweh D Witte J Function and metabolism of canine kidneys after aerobic ischemia by orthograde persufflation with gaseous oxygen. Res Exp Med (Berl). (1973) 159(4):288–97. 10.1007/bf01851603 4734455

[30] Isselhard W Denecke H Witte J Berger M Fischer JH. Renal function after hypothermic kidney ischemia with orthograde and retrograde O 2 -persulflation *in situ*. Res Exp Med (Berl). (1972) 157(3):231–4. 10.1007/bf01851149 5046308

[31] Isselhard W Witte J Denecke H Berger M Fischer JH Molzberger H. Function and metabolism of canine kidneys after aerobic ischemia by retrograde persufflation with gaseous oxygen. Res Exp Med (Berl). (1974) 164(1):35–44. 10.1007/bf01851962 4413518

[32] Sachweh D Isselhard W Dennecke H Stelter WJ Berger M Lauschke H Short time kidney preservation by hypothermic oxygen persufflation. Bull Soc Int Chir. (1972) 31(4):258–63. 4561449

[33] Papas KK Bellin MD Sutherland DE Suszynski TM Kitzmann JP Avgoustiniatos ES Islet oxygen consumption rate (ocr) dose predicts insulin independence in clinical islet autotransplantation. PLoS One. (2015) 10(8):e0134428. 10.1371/journal.pone.0134428 26258815 PMC4530873

[34] Papas KK Colton CK Nelson RA Rozak PR Avgoustiniatos ES Scott WE 3rd Human islet oxygen consumption rate and DNA measurements predict diabetes reversal in nude mice. Am J Transplant. (2007) 7(3):707–13. 10.1111/j.1600-6143.2006.01655.x 17229069 PMC2857994

[35] Papas KK Colton CK Qipo A Wu H Nelson RA Hering BJ Prediction of marginal mass required for successful islet transplantation. J Invest Surg. (2010) 23(1):28–34. 10.3109/08941930903410825 20233002 PMC3786417

[36] Papas KK Pisania A Wu H Weir GC Colton CK. A stirred microchamber for oxygen consumption rate measurements with pancreatic islets. Biotechnol Bioeng. (2007) 98(5):1071–82. 10.1002/bit.21486 17497731 PMC2859188

[37] Papas KK Suszynski TM Colton CK. Islet assessment for transplantation. Curr Opin Organ Transplant. (2009) 14(6):674–82. 10.1097/MOT.0b013e328332a489 19812494 PMC2859186

[38] Bunegin L Tolstykh GP Gelineau JF Cosimi AB Anderson LM. Oxygen consumption during oxygenated hypothermic perfusion as a measure of donor organ viability. ASAIO J. (2013) 59(4):427–32. 10.1097/MAT.0b013e318292e865 23820283

[39] Weegman BP Kirchner VA Scott WE 3rd Avgoustiniatos ES Suszynski TM Ferrer-Fabrega J Continuous real-time viability assessment of kidneys based on oxygen consumption. Transplant Proc. (2010) 42(6):2020–3. 10.1016/j.transproceed.2010.05.082 20692397 PMC2947551

[40] Annet L Hermoye L Peeters F Jamar F Dehoux JP Van Beers BE. Glomerular filtration rate: assessment with dynamic contrast-enhanced mri and a cortical-compartment model in the rabbit kidney. J Magn Reson Imaging. (2004) 20(5):843–9. 10.1002/jmri.20173 15503326

[41] Baumann D Rudin M. Quantitative assessment of rat kidney function by measuring the clearance of the contrast agent gd(dota) using dynamic mri. Magn Reson Imaging. (2000) 18(5):587–95. 10.1016/s0730-725x(00)00134-x 10913720

[42] Buchs JB Buehler L Moll S Ruttimann R Nastasi A Kasten J Dcd Pigs’ kidneys analyzed by mri to assess ex vivo their viability. Transplantation. (2014) 97(2):148–53. 10.1097/01.TP.0000438023.02751.22 24434482

[43] Tofts PS Cutajar M Mendichovszky IA Peters AM Gordon I. Precise measurement of renal filtration and vascular parameters using a two-compartment model for dynamic contrast-enhanced mri of the kidney gives realistic normal values. Eur Radiol. (2012) 22(6):1320–30. 10.1007/s00330-012-2382-9 22415410

[44] Kostidis S Bank JR Soonawala D Nevedomskaya E van Kooten C Mayboroda OA Urinary metabolites predict prolonged duration of delayed graft function in dcd kidney transplant recipients. Am J Transplant. (2019) 19(1):110–22. 10.1111/ajt.14941 29786954

[45] Castellino P DeFronzo RA. Glucose metabolism and the kidney. Semin Nephrol. (1990) 10(5):458–63. 2236987

[46] Cohen JJ Little JR. Lactate metabolism in the isolated perfused rat kidney: relations to renal function and gluconeogenesis. J Physiol. (1976) 255(2):399–414. 10.1113/jphysiol.1976.sp011286 1255526 PMC1309254

[47] Fischer W Manz F Schärer K. Renal substrate uptake, oxygen consumption and gluconeogenesis at low temperature. Curr Probl Clin Biochem. (1976) 6:65–73. 1001015

[48] Lee JB Peterhm. Effect of oxygen tension on glucose metabolism in rabbit kidney cortex and medulla. Am J Physiol. (1969) 217(5):1464–71. 10.1152/ajplegacy.1969.217.5.1464 5346315

[49] Vittorelli A Gauthier C Michoudet C Baverel G. Metabolic viability and pharmaco-toxicological reactivity of cryopreserved human precision-cut renal cortical slices. Toxicol In Vitro. (2004) 18(3):285–92. 10.1016/j.tiv.2003.08.010 15046775

[50] Wasung ME Chawla LS Madero M. Biomarkers of renal function, which and when? Clin Chim Acta. (2015) 438:350–7. 10.1016/j.cca.2014.08.039 25195004

[51] Pan HC Yang SY Chiou TT Shiao CC Wu CH Huang CT Comparative accuracy of biomarkers for the prediction of hospital-acquired acute kidney injury: a systematic review and meta-analysis. Crit Care. (2022) 26(1):349. 10.1186/s13054-022-04223-6 36371256 PMC9652605

